# Western corn rootworm (*Diabrotica virgifera virgifera*) transcriptome assembly and genomic analysis of population structure

**DOI:** 10.1186/1471-2164-15-195

**Published:** 2014-03-14

**Authors:** Lex E Flagel, Raman Bansal, Randall A Kerstetter, Mao Chen, Matthew Carroll, Ronald Flannagan, Thomas Clark, Barry S Goldman, Andy P Michel

**Affiliations:** Monsanto Company, 700 Chesterfield Parkway W, Chesterfield, MO 63017 USA; Department of Entomology, Ohio Agricultural Research and Development Center, 1680 Madison Ave., Wooster, OH 44691 USA

**Keywords:** *Diabrotica virgifera virgifera*, Insect resistance, Transgenic crop, Transcriptome, Western corn rootworm

## Abstract

**Background:**

Western corn rootworm (WCR) is one of the most significant insect pests of maize in North America. WCR has dramatically increased its range in the last century, invading key maize production areas in the US and abroad. In addition, this species has a history of evolving traits that allow it to escape various control options. Improved genetic and genomic resources are crucial tools for understanding population history and the genetic basis of trait evolution. Here we produce and analyze a transcriptome assembly for WCR. We also perform whole genome population resequencing, and combine these resources to better understand the evolutionary history of WCR.

**Results:**

The WCR transcriptome assembly presented here contains approximately 16,000 unigenes, many of which have high similarity to other insect species. Among these unigenes we found several gene families that have been implicated in insecticide resistance in other species. We also identified over 500,000 unigene based SNPs among 26 WCR populations. We used these SNPs to scan for outliers among the candidate genes, and to understand how population processes have shaped genetic variation in this species.

**Conclusions:**

This study highlights the utility of transcriptomic and genomic resources as foundational tools for dealing with highly adaptive pest species. Using these tools we identified candidate gene families for insecticide resistance and reveal aspects of WCR population history in light of the species’ recent range expansion.

**Electronic supplementary material:**

The online version of this article (doi:10.1186/1471-2164-15-195) contains supplementary material, which is available to authorized users.

## Background

Western corn rootworm (*Diabrotica virgifera virgifera*) is a beetle (order Coleoptera) that is native to North America and the dominant maize pest in the US Corn Belt. It is estimated that each year Western Corn Rootworm (WCR) costs US farmers at least 1 billion dollars through yield losses and treatment costs [1, 2]. WCR is native to North America, being first described in the western Kansas in 1867 [3]. Throughout the 20^th^ century it spread eastward, colonizing the major maize production areas in the north central US (the Corn Belt) [3]. More recently it invaded Europe [3, 4], making it now a global pest of maize production.

The impact of WCR on maize production has been exacerbated by the fact that WCR has proved difficult to control. Over the last few decades WCR has evolved resistance to crop rotation [3, 5, 6], a range of chemical insecticides [7–10] and to transgenic maize expressing the Cry3Bb1 protein from *Bacillus thuringiensis* (Bt) [11]. The genetics behind some WCR resistance mechanisms have been investigated [12–14]. For example, a single nucleotide polymorphism (SNP) in the gamma-aminobutyric acid (GABA) receptor is correlated with cylcodiene resistance [14]. However, for many of the most pressing resistance traits the genetic basis remains unknown.

Developing a genetic toolbox for WCR is an important step toward better understanding the genetic basis of insecticide resistance in WCR. To this end, Siegfried et al. [15] produced 691 expressed sequence tags (ESTs) from WCR midgut tissues and screened them for candidate receptors for Bt. Among these ESTs, they reported one cadherin (a putative Bt receptor) and 15 cathespin-like fragments (cysteine proteases) that are potential candidates for resistance to Bt and other toxins.

Studies in other insect species have suggested that cytochrome P450s (CYPs) are involved in resistance to several insecticides including methyl parathion, carbaryl, carbamate and organophosphates (Scharf et al. [16] and references therein; [17, 18]). In WCR only 3 CYPs have been characterized, and all belong to the CYP4 clade, which is not typically associated with insecticide resistance [16]. Furthermore, a variety of other candidate genes for insecticide or Bt resistance are lacking for WCR, including esterases and other receptors [19, 20]. A more complete understanding of the diversity of these important resistance associated gene families can lead to a more rapid characterization of current and future resistance mechanisms.

In addition to resistance, questions remain regarding WCR population structure and patterns of gene flow. Previous studies for North American WCR populations have used several types of molecular markers (allozymes, mtDNA sequencing, AFLPs, microsatellites, and SNPs), with all studies indicating large amounts of genetic diversity and a general lack of population structure across the Corn Belt [10, 21–25]. These findings are consistent with the hypothesis that WCR has retained large amounts of genetic diversity during the species’ rapid eastward range expansion. Whether or not this pattern is shared across the entire WCR genome or if certain areas contain less genetic diversity is unknown.

As the amount gene flow among populations largely dictates the spread of resistance alleles, a better understanding of the genomic variation across populations will lead to a more informed assessment of resistance monitoring and mitigation, and prolong the life of various control options. Modern DNA sequencing technologies have increased our ability to undertake large-scale sequencing projects for almost any organism, including those with complicated genomes such as WCR [26]. Therefore, our first objective for this study was to characterize the WCR transcriptome using publically available data as well as new data generated by *454* Roche pyrosequencing. We then mined this data to identify candidate genes in pathways predicted to be involved in insecticide resistance. Our second objective was to develop an expansive set of SNPs that can be used for more robust inferences of population structure, construction of linkage maps, and genomic scans of selection. To produce these SNPs we generated genomic sequences from multiple laboratory and field populations, including five populations that showed reduced response to Cry3Bb1 in lab-based single plant bioassays. Furthermore, our ability to combine both transcriptomic and genomic data provide important information regarding genetic variation and level of selection among candidate resistance genes.

## Methods

### Sample collection, EST sequencing and assembly

For *454* EST sequencing WCR third-instar midgut tissues were isolated from 200 larvae from the Waterman population (Table 1) and stored in RNAlater (Life Technologies) prior to extraction. Total RNA extraction was performed on four sets of 50 midguts using the RNeasy mini kit (Qiagen). Approximately 500 μg of total RNA were used for two rounds of PolyA selection using the MicroPoly(A)Purist kit (Life Technologies). Following PolyA selection, cDNAs were synthesized using the SuperScript II cDNA synthesis kit (Life Technologies), using random hexamers for priming during first-strand synthesis. Finally, cDNAs were purified using a QIAquick PCR purification column (Qiagen).

**Table 1:** **Population details for all 26 populations sampled in this study**

Population name	State	Year	Source	Diapause	Response to Cry3Bb1	π
Jones	IA	2011	Field	Yes	NA	0.0064
Plainview	MN	2011	Field	Yes	NA	0.0063
Gayville	SD	2011	Field	Yes	medium	0.0067
Humphrey	NE	2011	Field	Yes	high	0.0061
Sherman 2	CO	2011	Field	Yes	NA	0.0063
North Mankato	MN	2011	Field	Yes	medium	0.0065
Maynard	IA	2011	Field	Yes	NA	0.0064
Kit Carson 2	CO	2011	Field	Yes	high	0.0063
Onslow	IA	2011	Field	Yes	low	0.0066
Kit Carson 3	CO	2011	Field	Yes	medium	0.0065
Shell rock	IA	2011	Field	Yes	medium	0.0063
Trent	SD	2011	Field	Yes	low	0.0063
Adams	IN	2012	Field	Yes	high	0.0058
Fillmore	MN	2012	Field	Yes	NA	0.0057
Colfax	NE	2012	Field	Yes	NA	0.0059
Moody	SD	2012	Field	Yes	low	0.0057
Sherman 1	KS	2012	Field	Yes	medium	0.0058
Hansel	IA	2012	Field	Yes	NA	0.0059
Kit Carson 1	CO	2012	Field	Yes	NA	0.0059
Knox	IL	2012	Field	Yes	medium	0.0057
Hopkinton	IA	2009	Lab	No	low	0.0056
Brookings (random mated)	SD	NA	Lab	No	high	0.0055
Brookings (inbred)	SD	NA	Lab	No	high	0.005
Seneca resistant	KS	2005	Lab	No	low	0.0056
Seneca susceptible	KS	2005	Lab	No	NA	0.0056
Waterman	IL	NA	Lab	Yes	high	0.0063

For each population the state of origin, year collected, source, diapause status, response to Cry3Bb1, and intra-population genetic diversity (π) are given. Diapause status indicates whether or not the population undergoes diapause. All non-diapause (“No”) populations derive this phenotype from the Brookings, SD source. All field sourced populations diapause. Response to Cry3Bb1 was determined by larval recovery data in single plant bioassays under laboratory conditions. Each population was reared on Cry3Bb1 expressing (treatment) and isoline (control) maize. Populations with at least 80% relative survival on treatment when compared to control were defined as low response, while medium response populations ranged between 20% and 80%, and high response populations were less than 20% relative survival. Missing data is indicated by NA.

Before *454*-FLX sequencing, emulsion PCR reactions were conducted following the manufacturers specifications (Roche). In total, 1,271,800 *454* EST reads were generated with a mean length of 297.6 bp, together totaling 378,442,184 bp of total sequence. These *454* sequences were supplemented with 17,833 Sanger sequences from NCBI (sequence IDs provided in Additional file 1) adding an additional 9,331,393 bp. The Sanger sequences derive from a variety of tissues and life cycle stages, including many previously published by Siegfried et al. [15].

Prior to assembly all *454* sequences were trimmed of their adaptors, while all Sanger reads were trimmed by searching against the NCBI UniVec database of common cloning vectors. *454* and Sanger sequences were merged and assembled with Newbler 2.3. All *454* ESTs can be found on the NCBI Sequence Read Archive (Accession SRS528965).

### Transcriptome annotation and characterization

The WCR transcriptome was annotated using Blast2GO [27]. BLASTx searches (*e* value < 10^-3^) were performed between WCR unigene sequences and the NCBI non-redundant (nr) database. Following the mapping step, gene ontology (GO) terms with *e* value < 10^-6^, annotation cut-off > 55, and GO weight > 5 were used for annotation. The “Aqua” classification in CateGOrizer [28] was used to categorize the GO terms into different GO categories. The GO categories for WCR were compared to those from *Tribolium casteneum* (available at http://www.b2gfar.org/showspecies?species=7070). To find the pathways in which putative proteins of the WCR transcriptome are involved, analysis of Kyoto Encyclopedia of Genes and Genomes (KEGG) was performed using Blast2GO [29]. For comparative genomics, pairwise BLASTx searches (*e* value < 10^-3^) between WCR contig sequences and genomes of model insect species (*Bombyx mori, Drosophila melanogaster, Nasonia vitripennis, Tribolium casteneum*) were performed. Results of these BLASTx searches were also used to calculate the ortholog hit ratio ([30]; at OARDC MCIC galaxy: http://www.oardc.ohiostate.edu/mcic/bioinformatics/bionformatics.html). The ortholog hit ratio was calculated by dividing the number of non-gap characters in the query (WCR unigene) by the length of the subject (model insect ortholog) [30].

### Pooled population resequencing and SNP detection

**Figure 1:**
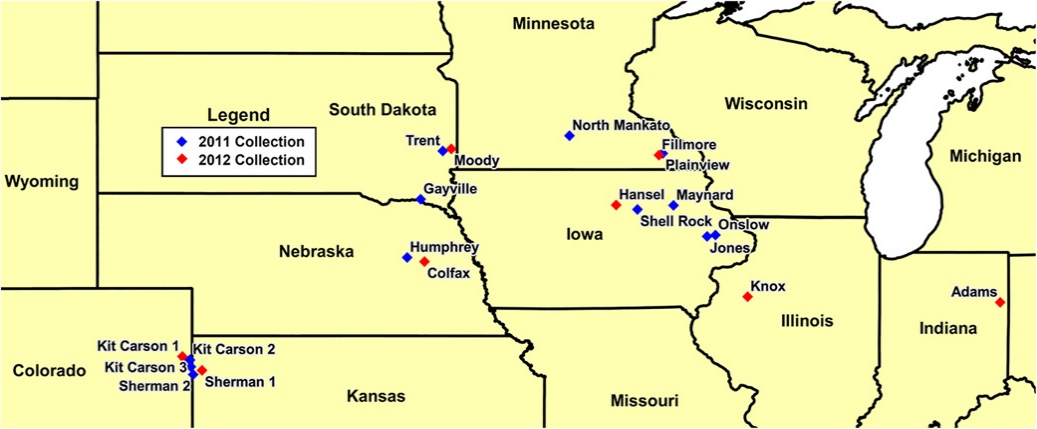
**Map of WCR field locations.** Collection locations are given for the 20 field collected populations used in this study. 2011 collections are marked in blue, while 2012 collections are marked in red.

Population genomic diversity was estimated by sampling 26 WCR populations (Figure 1; Table 1), 20 of which originated from field collections made in 2011 and 2012 (to protect the anonymity of the landowners only approximate locations are given for field collections). Where available, we also list each population’s phenotypic status for diapause and response to the Cry3Bb1 Bt toxin based on single plant bioassays under laboratory conditions [11] (M. Chen et al., in prep). The remaining 6 populations originated from both diapause and non-diapause laboratory colonies. Among these populations the Hopkinton and Seneca research colonies are resistant to Cry3Bb1 [11, 31].

For each population we randomly selected 5 individuals (effectively 10 haploid chromosome sets) from mixed sex populations for Illumina sequencing (130 total individuals). All individuals within a population were combined prior to DNA extraction (5 individuals per pool). DNA was extracted using the E.Z.N.A. Insect DNA isolation kit (Omega Bio-Tek). Illumina sequencing libraries were prepared using the Nextera kit (Illumina) and sequenced on an Illumina HiSeq 2000 genome sequencer in a 2 × 100 bp paired-end configuration. After sequencing, barcode adapters were removed and all reads were quality trimmed from both ends to a minimum PHRED score of 20. All raw Illumina sequences used in this study can be found on the NCBI Sequence Read Archive (Accession SRP035322).

Paired-end Illumina genomic shotgun sequences from all populations were aligned to the combined transcriptome assembly described above using BWA (version 0.5.8c; [32]), with default settings. Aligning genomic sequences to a reference transcriptome creates mismatches at exon/intron boundaries because the introns are sampled by the genomic sequences but typically spliced out of the transcripts. When BWA encounters these mismatches it issues a “soft-clip” flag [33]. To avoid SNP calling errors that might be associated with these mismatches, all reads were first filtered to remove any reads that had been soft-clipped. We also removed all reads with a “MAPQ” alignment score < 25. Finally, SNPs were extracted from these alignments using the *mpileup* program from the SAMtools package (version 0.1.18; [33]), at all sites with at least 5X coverage from nucleotides with a PHRED sequence quality ≥ 30. All SNP calls can be found in Additional file 2.

To validate SNPs, we used the Cleaved Amplified Polymorphic Sequences (CAPS) technique [34]. Potential CAPS markers were identified *in silico* by screening alternate SNP haplotypes against the commercially available restriction enzyme database found in Biopython (version 1.60; [35]). We selected 27 SNPs that created a polymorphism in a recognition site for the enzyme *RsaI*. A region around each SNP was amplified and digested in up to 11 WCR individuals (average 9 per marker). Primer sequences can be found in Additional file 3. PCR reactions were performed in a 20 μl mixture containing 1 μl (50 ng/μl) DNA template, 10 μl 2X PCR Promega master mix (with a final concentration of 0.4 mM each deoxynucleoside triphosphate, 1.5 mM MgCl2 and 0.625 units of *Taq* DNA polymerase in PCR reaction buffer pH 8.5) and 0.5 mM each primer. Amplified products for each sample were digested with 1 μl (10 u/μl) *RsaI* at 37°C for 2 hrs. The resulting digest was visualized after electrophoresing in a 2% agarose gel stained with ethidium bromide.

### Estimating population genetic parameters

We estimated nucleotide diversity within populations for each unigene using a bias-corrected estimator (; simply *π* throughout this manuscript) derived for pooled populations of individuals [36]. This estimator requires a minimum count (*b*) for the inclusion of a particular SNP variant in the calculation. Here we used *b* = 2 as our minimum. We also disregarded any sites with greater than > 40X coverage to minimize the inclusion of paralogous sequences or undetected copy number variants. Within each population mean *π* was approximately 0.006, however in each population we observed 16 to 30 outlier unigenes (*π* > 0.15). These outliers were removed prior to the calculation of summary statistics, as these loci are likely impacted by alignment issues given their extreme departure from the mean. These calculations were done using a custom Python script (available on request).

We computed pairwise *F*_ST_ between all populations for each unigene using a bias-corrected estimator for pooled population samples as implemented in PoPoolation2 (version 1.201; [37]), again using a minimum coverage of 5 and a maximum coverage of 40 for each SNP locus. Window and step sizes were both set to 10^9^, forcing the program to estimate *F*_ST_ once for each unigene.

*F*_ST_ is a measure of genetic differentiation relative to the net genetic diversity of the populations sampled, which makes *F*_ST_ sensitive to fluctuations in net genetic diversity. To complement *F*_ST_, several authors have concluded that absolute metrics of genetic differentiation should also be employed [38, 39]. For this study we created a simple statistic (*π*_*xy*_) to estimate absolute genetic differentiation between populations using pooled sequence data. This statistic is an estimator of Nei’s *d*_*XY*_ statistic [40], though unlike *d*_*XY*_ it uses estimated allele frequencies rather than allele counts, making it suitable for pooled sequence data. To estimate *π*_*xy*_ we first recorded a four element sequence profile *b*_*1*_, *b*_*2*_*, b*_*3*_*, b*_*4*_, for every nucleotide site within each pooled population, that contains the frequency of A, C, G, and T bases in the sample. Then for each unigene we calculated the average per-site expected heterozygosity among gametic unions between populations *x* and *y* (i.e. *π*_*xy*_), using the equation below.


Where *n* is the number of nucleotide sites available for a unigene,  and  are the frequencies of base *b*_*j*_ at nucleotide site *i* in populations *x* and *y*, respectively. *π*_*xy*_ requires only estimates of within population allele frequencies and not estimates of within population nucleotide diversity, unlike *π* and *F*_ST_, and thus *π*_*xy*_ does not require bias-correction for pooled populations.

We applied the *π*_*xy*_ metric to calculate genetic distances between populations for principal coordinates analysis (i.e. classical multidimensional scaling). We also applied the *π*_*xy*_ metric to characterize the genetic divergence between combined populations with low or high responsiveness to Cry3Bb1 or diapause and non-diapause populations (Table 1). To estimate *π*_*xy*_ from combined populations we used the average of the nucleotide frequencies within each population to avoid biases that could arise due to uneven sequence coverage between populations. As with *π*, we required a minimum coverage of 5 and a maximum coverage of 40 within each population for a site to be considered. We also required a minimum count of 2 for the inclusion of a particular SNP variant.

For each unigene we identified *π*_*xy*_ and *F*_ST_ outliers by permuting population membership and calculating both metrics for permuted pairs. In the case of the Cry3Bb1 response phenotype there are  possible permutations between high and low response populations, and all permutations were used to create the null distribution. For diapause versus non-diapause populations there are  potential permutations, among which we randomly selected 1,000 to create the null distribution. In either case we selected the 99^th^ percentile *π*_*xy*_ and *F*_ST_ values from among these permuted populations as our threshold for significance, after removing all unigenes with fewer than 50 available nucleotide sites. This threshold is arbitrary and lacks multiple test correction, though our goal is to produce a list of potential candidates that is inclusive, though it may include some false positives. Also, given the permutation tests outlined above, the most confidence one can have regarding significance is 1/462 or 1/1,000, respectively.

Our sampling procedure involved estimating population parameters from five individuals per population across approx. 10,000 loci. Theory suggests that multi-locus estimates of population genetic parameters can be robust even when estimated from just five individuals [41, 42]. Though this theory applies specifically to direct sequencing of individuals and not pooled population sequencing, we have conducted a power analysis (Additional file 4) of our pooled sampling design, and it confirms that adequate estimates can be made from just five individuals, especially for statistics utilizing the grand mean across all loci. In light of this, our grand mean genomic estimates (i.e. mean within population *π* and between population *F*_ST_) should be quite accurate, despite the small within population sample size. Due to the small within population sample size (n = 5), we expect our single locus estimates of divergence (*i.e.* the *π*_*xy*_ and *F*_ST_ outlier scans described above) to have relatively modest statistical power. These scans should be viewed as exploratory.

## Results and discussion

### *De novo*assembly and annotation

**Figure 2:**
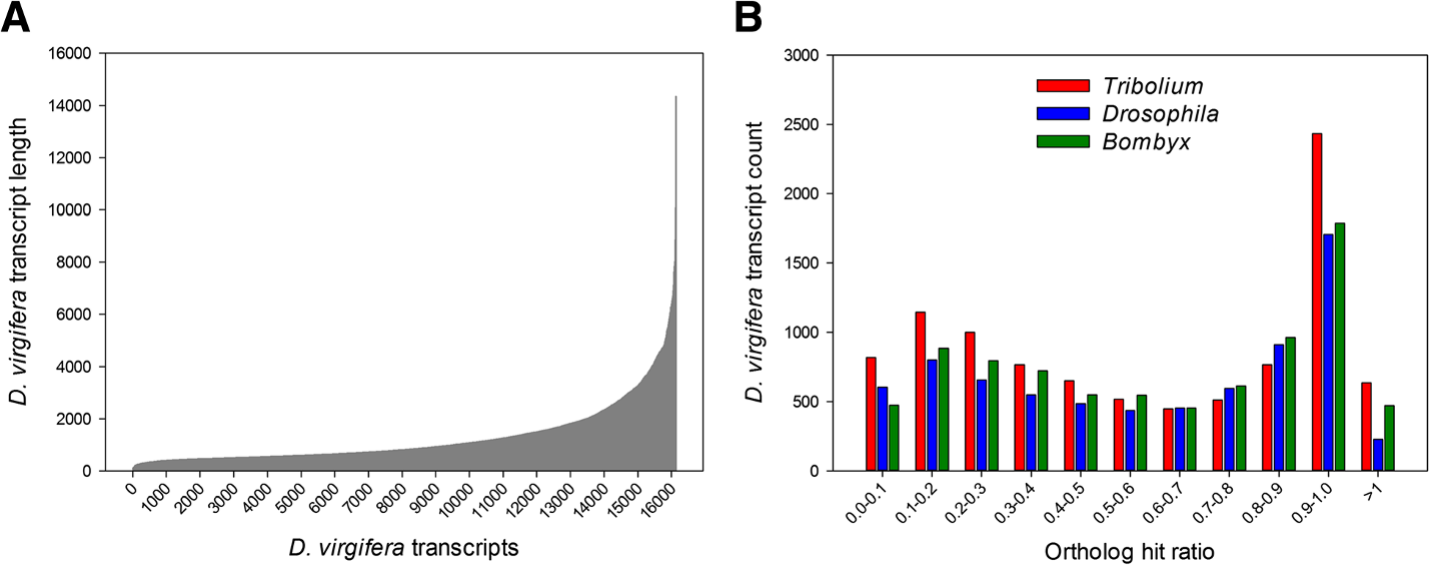
**Transcriptome assembly of WCR. A)** Length distribution of all 16,130 transcripts; each transcript is placed along x-axis in the ascending order based on its length, and **B)** ortholog hit ratio for assembled contigs calculated after BLASTx searches to genomes of *T. casteneum*, *D. melanogaster*, and *B. mori*.

The *de novo* assembly of the WCR transcriptome yielded 16,130 high quality unigenes totaling 20,643,715 bp, in addition to 95,335 singleton reads. The length of unigenes varied from 99-14,348 bp with an average of 1,280 bp (Figure 2A). To determine the completeness of WCR transcriptome assembly, each unigene was compared to its putative ortholog from *T. casteneum, D. melanogaster*, and *B. mori*. Overall, 44-46% of the unigenes (with matches) had an ortholog hit ratio > 0.7 and 54-58% were > 0.5 (Figure 2B). On the assumption of conserved gene length between species, an ortholog hit ratio near zero indicates a poor assembly while values near one indicate a fully assembled transcriptome [30]. Following these criteria the current assembly for WCR appears to be moderately complete.

About 64% (10,359/16,130) of the WCR unigenes had one or more hits to protein sequences in the non-redundant (nr) database at GenBank while the remaining (36%) had no match (Additional file 5). The majority of top blast hits for WCR unigenes were to insects (~93%), whereas a small proportion showed top hits to non-insect arthropods (~1%), other eukaryotes (~5%), and bacteria (< 1%). As expected, more than two-thirds of the top hits for the WCR unigenes were to coleopteran sequences in the nr database such as those of *T. casteneum*, *Dendroctonus ponderosae*, *Chrysomela tremula*, *Leptinotarsa decemlineata*, *Phaedon cochleariae*, *Tenebrio molitor*, and *Batocera horsfieldi*. The WCR unigenes with no match to the nr database (unmatched) may represent novel genes with functions not yet been identified, or they could be a product of assembly errors. Interestingly, the percentage of unmatched unigenes obtained in our study is considerably lower than those obtained for other insect transcriptomes [43–45]. An InterProScan search [46] of these unmatched WCR unigenes against the nr database revealed hits to the protein signature databases for 2,357 out of 5,762 (41%), suggesting that many have homologs in other species that were undetected using BLASTx alone. Further, 1,423 of the unmatched unigenes had an open reading frame (ORF) of at least 50% of the contig length and 302 had an ORF of at least 90% of the contig length. A pairwise comparison to the recently sequenced genome of mountain pine beetle [47] did not improve the annotation significantly as only 132 out of 5,762 unmatched WCR unigenes showed hits (data not shown).

### Comparative genomics

Using pairwise BLASTx searches to protein databases for four model insects, significant matches for a majority of WCR unigenes (10,356/16,130) were obtained. The BLASTx search to the *T. casteneum* database showed the highest number of matches for WCR unigenes (n = 9,693), followed by searches to *N. vitripennis* (n = 8,981), *B. mori* (n = 8,257), and *D. melanogaster* (n = 7,422) databases (Additional file 6). Several WCR unigenes (n = 6,767) showed matches to all four databases. However, there were a substantial number of WCR unigenes that matched uniquely to *T. casteneum* (n = 754), *N. vitripennis* (n = 288) and *B. mori* (n = 130). Analyzing the pairwise comparisons, the highest level of agreement was observed between WCR and *T. casteneum*, likely reflecting the evolutionary relatedness between these two insect species and the completeness of the *Tribolium* genome [48].

### Functional annotation

Using Blast2GO, about 46% of (7,377/16,130) WCR unigenes were annotated. The observed gene ontology (GO) terms for each domain (biological process, molecular function and cellular component) were widely distributed into different categories (Additional file 7). A comparison of percent mappings to each GO category in WCR and *T. casteneum* revealed a highly similar distribution for both insect species. The majority of contigs assigned to the ‘biological process’ domain were involved in cellular, regulatory, and developmental activities, while most of the contigs under the ‘molecular function’ domain were predicted to have catalytic, binding and transporter functions. Through the KEGG-based pathway analysis using Blast2GO, a total of 2,376 WCR unigenes were assigned to one or more of 117 pathways (Additional file 8). The majority of contig sequences were assigned to pathways for metabolism of organic compounds such as purine, pyrimidine, and glucose. The information on predicted pathways coupled with the functional annotation of the WCR transcriptome provides useful information for future genetic studies in this insect.

We further characterized potential genes that may be involved in WCR Bt resistance, specifically to Cry3Bb1 [11], focusing on detoxification and degradation (cytochrome P450s (CYPs), esterases, and cathepsins). We also identified several unigenes identified as putative receptors for Bt toxins [20, 49–53] such as cadherins, ABC transporters, aminopeptidases and glycosyltransferases. (Note: glycosyltransferases are not technically themselves receptors, but are hypothesized to be responsible for glycosylation of lipids which functions as receptors [52].) Additional file 9 lists the WCR unigenes that significantly matched these sequences in other insects, mainly from *Tribolium*. The number of putative receptors totaled 32, whereas the number of cathepsins, CYPs, and esterases were much larger (n = 98, 90, and 70, respectively). These totals mark a considerable expansion beyond what was previously known for WCR candidate resistance gene targets. We further characterized two of the more important groups, the CYPs and cathepsins, because of their known relevance to resistance.

Cytochrome P450s (CYPs) are a superfamily of enzymes which are ubiquitous among organisms and play an important role in metabolizing endogenous and exogenous substances. In insects, CYP genes are classified into four clades: CYP2, CYP3, CYP4, and mitochondrial P450s [17]. Despite their importance for insecticide resistance in WCR and other insects [17, 18], little information is known regarding the diversity or genetic variation of WCR-CYPs. In addition to BLASTx searches against the NCBI nr database, we identified the putative clades and families of WCR-CYPs through a BLASTp comparison to a recently published *Tribolium* CYP database [18]. The majority of WCR-CYPs had highest similarity to clade 3 (n = 65), with a moderate number (n = 19) showing similarity to clade 4 (Additional file 10).

The expanded *454* sequencing combined with previous resources show a considerable diversity in the number of cathepsins in WCR. We found a total of 98 unigenes that matched known cathepsins, including some with high similarity to those already described in WCR [15] (Additional file 9). A previous EST-based sequencing project [15], revealed 15 cathepsin-like fragments (a total of 171 sequences that assembled into 11 contigs and 4 singletons), corresponding to two different classes, L and B. In our transcriptome data, 18 unigenes belonged to class B and 55 matched class L; we also identified 8 unigenes in the lesser known D and F classes (N = 6 and N = 2, respectively, Additional file 9). Cathepsins play a pivotal role in WCR digestion, and have been implicated in the evolution of rotation resistance. Normally, WCR feed and oviposit in corn, but rotation resistant WCR feed on soybean before oviposition. When these soybean fields are rotated to corn the following year, the rotation resistant WCR eggs hatch and larvae feed on emerging corn plants. Curzi et al. [13] found that WCR feeding on soybean leaves had higher levels of cathepsin-L like activity compared to WCR fed on corn. This expanded set of cathepsin-L like sequences and targeted resequencing of WCR populations could help enable the development of a molecular diagnostic for rotation resistant WCR.

**Figure 3:**
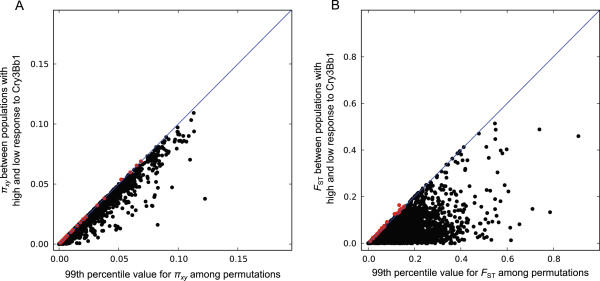
**Calculated** ***π***_***xy***_ **and** ***F***_**ST**_ **values between populations with high and low response to Cry3Bb1 versus permuted 99** ^**th**^ **percentile values.** Each point represents a unigene’s calculated *π*_*xy*_ **(A)** or *F*_ST_ **(B)** value (*y*-axis) between pooled high and low response to Cry3Bb1 populations and the 99^th^ percentile value from all possible permutations of these populations (*x*-axis). Red dots indicate calculated *π*_*xy*_ or *F*_ST_ values > 99^th^ percentile permutation value (outliers), while black dots represent calculated *π*_*xy*_ or *F*_ST_ values < 99^th^ percentile permutation value. The blue line represents the boundary between outliers and non-outliers.

For all unigenes, including those that may be involved in WCR resistance to Cry3Bb1, we pooled SNP data from different populations with high or low response to Cry3Bb1 (Table 1) and screened for genes that were *π*_*xy*_ and *F*_ST_ outliers (see Methods). We identified 39 *π*_*xy*_ outliers and 26 *F*_ST_ outliers (Figure 3; Additional file 9). Only one unigene was found on both lists (unigene: DIAVI-09JAN12-CLUS09972_1). This unigene shares sequence similarity with a gene of unknown function from *T. castaneum* (GenBank ID: XP_976004). Among all 64 *π*_*xy*_ and *F*_ST_ outlier unigenes we found two on our lists of candidates for genes potentially involved in reduced response to Cry3Bb1. These include one esterase (unigene: DIAVI-09JAN12-CLUS09401_1) and one CYP (unigene: DIAVI-09JAN12-CLUS02697_1; clade 3, CYP9), both of which were *F*_ST_ outliers. All outliers should be considered preliminary and exploratory as our tests are low-powered given the relatively small within population sample sizes, and because our strategy assumes that populations with a low response to Cry3Bb1 share resistance alleles, though there is no evidence supporting this assumption. Nonetheless, these loci may represent a promising pool of potential candidates for Cry3Bb1 resistance.

### WCR SNP development and validation

**Figure 4:**
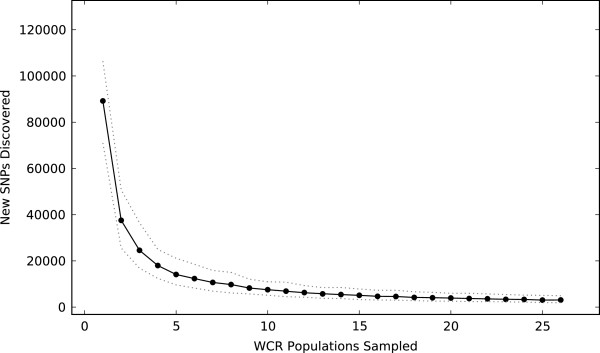
**Rarefaction curve of SNP discovery among the 26 WCR populations sampled.** Starting with the first population sampled and continuing until the 26^th^ we record the number of new SNPs each population contributes. The slope of the rarefaction curve is dependent on population input order, which in this case is arbitrary. To account for this, we selected 100 random input orders and computed the mean number of new SNPs discovered (solid black line with filled points) and the upper and lower 95% confidence intervals (dashed grey lines).

The field collected populations (Figure 1) included a wide geography across the US Corn Belt, while the laboratory populations (Table 1) included several common research strains. Each population consisted of 5 individuals (effectively 10 haploid chromosome sets) that were pooled and sequenced to approximately 20X-30X coverage using whole genome shotgun sequencing. These sequences were aligned to the reference transcriptome for SNP discovery and estimation of population genetic parameters. In total we found 532,396 SNPs segregating among the 260 haploid chromosome sets sampled. SNP discovery saturates quickly among the WCR populations we sampled (Figure 4). After sampling 5 populations, few new SNPs are detected among the remaining populations. Those new SNPs that are found by adding more than 5 populations tend to be rare, often occurring in just one population. Thus, within our unigene collection, it is possible that our data has revealed most of the common SNPs in Corn Belt WCR.

To address the validity of our SNP predictions and their utility for genotyping we identified 27 that were predicted to create a polymorphic restriction enzyme site and used this to genotype individuals from the inbred and randomly mated Brookings population using CAPs (see Methods). For 24 of the 27 SNPs the CAPs genotypes showed evidence of both allelic versions of the predicted SNP (Additional file 3). The remaining 3 SNPs were homozygous for one allele among all individuals tested, which could reflect inaccurate SNP predictions or simply the absence of one allele among the populations tested. In any case, these results demonstrate that the predicted SNPs are reliable and can be readily utilized in genotyping assays.

### Population genomics of western corn rootworm

Using the SNPs extracted from pooled population sequencing we estimated genetic diversity within and between WCR populations. To estimate within population nucleotide diversity (*π*) we used a bias-corrected estimator [36], suitable for pooled population sequences. For between population comparisons we created a simple statistic (*π*_*xy*_), which is an estimator of the *d*_*XY*_ statistic [40]. We focused our analyses on the 10,359 WCR unigenes with a hit to a protein in the NCBI nr database (300,425 SNPs). For these unigenes, the mean *π* within each population can be found in Table 1. Nucleotide diversity within populations ranged from 0.005-0.007, with a mean of 0.006. As expected, the inbred Brookings colony (5 generations of full-sib mating; Chad Nielson pers. comm.) showed the lowest mean *π*, and the highest mean *π* values were found among field collected populations.

Our extensive SNP dataset reveals several interesting aspects of genetic variation in WCR. First, the inbred population had experienced 5 generations of full-sib mating, which is theoretically expected to fix approximately 41% of the sites segregating among the gametes of the founding sib-mated pair (Table 5.1 in Falconer and Mackay [54]), yet we observed only a slight reduction in *π* within the inbred Brookings population (mean *π* = 0.005) compared to the randomly mated Brookings population (mean *π* = 0.0055) from which it was derived. This suggests that full-sib mating has been fairly ineffective at removing variation. Second, there appears to be slightly less nucleotide diversity within the 2012 collections, when compared to the 2011 collections (t-test *p*-value < 0.001). This could reflect annual variation in population sizes across the Corn Belt, or perhaps some unnoticed deviation in our sampling procedures between these two years. Finally, there is little evidence for a significant reduction of genetic diversity among lab populations, suggesting that maintenance in artificial environments has not caused an erosion of genetic diversity. This finding mirrors observations by Kim et al. [55], who compared a laboratory population derived from the non-diapause Brookings population to field populations using microsatellite markers.

Among the 325 pairwise comparisons between populations, *π*_*xy*_ ranged between 0.0079 and 0.0097 with a mean of 0.0088. From this we observed that the levels of within population nucleotide diversity (mean *π* = 0.006) are of a similar magnitude as between population divergence (mean *π*_*xy*_ = 0.0088). This indicates that each population maintains considerable genetic variation, and that there is little evidence for substantial genetic differentiation between populations (see below).

**Figure 5:**
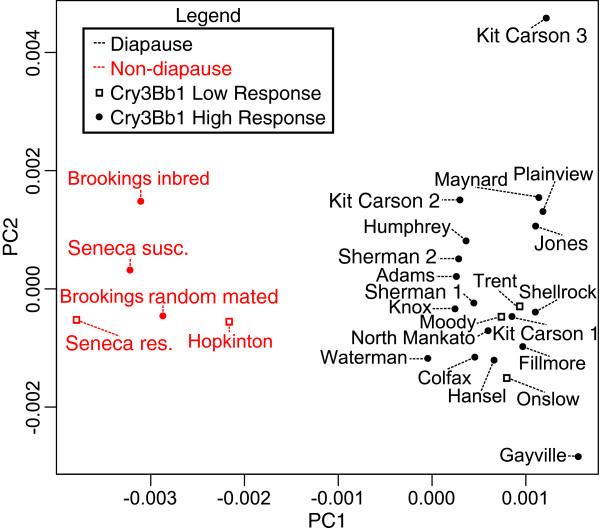
**Principal coordinate analysis using genetic distances between 26 WCR populations.** Diapause populations are colored in black and non-diapause population are red. Populations with a reduced response to Cry3Bb1 are denoted with open squares, while populations with unknown or low to intermediate resistance are denoted with filled circles.

Pairwise *π*_*xy*_ values were also used to generate a principal coordinates analysis among all populations. Diapause status appears to be the most important factor in population differentiation (Figure 5). The natural state for WCR is diapause, and the trait shared by all 5 non-diapause populations was inherited from a single source [56]. This non-diapause trait was developed after several generations of mass selection, and because of its complex inheritance, it has been suggested that it is likely a complex polygenic trait [56]. Consistent with this hypothesized mode of inheritance and strong artificial selection for its maintenance, we find 253 unigenes that are *π*_*xy*_ outliers when comparing all diapause versus all non-diapause populations (Additional file 11 and Additional file 12; see Methods). These outlier unigenes may include causal alleles for the non-diapause phenotype, but likely also include linked loci that have been differentiated between diapause and non-diapause populations by genetic hitchhiking.

To more formally address population differentiation we estimated *F*_ST_ pairwise between all populations for all 10,359 unigenes with a hit to the NCBI nr database. Mean *F*_ST_ between all populations across all unigenes was 0.052 (95^th^ percentile bootstrap confidence intervals [0.051, 0.053]), and only 5.6% of all unigenes had *F*_ST_ values > 0.2 (Additional file 13). The overall pattern of low *F*_ST_ indicates little genetic differentiation among WCR populations, even across distances of approximately 1,500 km. These findings are consistent with earlier work in WCR, which also found low levels of genetic differentiation between field collected populations [22–25]. They are also consistent with our observation that new SNP discovery saturates fairly quickly as new Corn Belt populations are sampled (Figure 4).

Among all pairwise population contrasts, the highest *F*_ST_ values were found between field and lab populations, with nearly all of the most extreme values between a field population and the inbred Brookings population (data not shown). This observation is expected, as inbreeding impacts allele frequencies and will tend to increase *F*_ST_. Interestingly, the smallest mean pairwise *F*_ST_ estimate was between the Onslow, IA and Trent, SD populations (mean *F*_ST_ = 0.029; 95^th^ percentile bootstrap confidence intervals [0.028, 0.030]). Despite being 508 km apart, these two field collected populations have both demonstrated reduced response to Cry3B1 in a lab-based single plant bioassays (Table 1). It is premature to conclude that these populations derive from a single Cry3Bb1 resistant source, though this intriguing possibility warrants further research.

In the last 75 years WCR have expanded from their native range in the US Southwest into the Corn Belt and further eastward, recently arriving at the Eastern Seaboard [3]. Given this recent and rapid range expansion, it is not surprising that our results and previous efforts [22–25] reveal limited genetic differentiation among Corn Belt WCR populations, even across distances of approximately 1,500 km. Theoretical treatments of the genetic properties of range expansion typically find that populations near the edges of the range have reduced genetic diversity and increased *F*_ST_ when compared to populations near the center of the range, and that both of these features reset after colonization at a rate that is proportional to the migration rate between populations (reviewed by Excoffier et al. [57]). Among the Corn Belt WCR populations, we find high genetic variation and low *F*_ST_ values among the most recently colonized easterly populations sampled (Knox, IL and Adams, IN; likely colonized < 40 years ago [3]). These populations are now over 1,000 km west of the eastern edge of the species range, and our genetic observations are consistent with theoretical predictions, in that these populations are no longer in their colonizing phase and are likely gaining new genetic diversity through gene flow with more westerly populations. This pattern may also suggest that migration within the Corn Belt is fairly high; enough to spread considerable genetic diversity into populations founded in the last 40 years. Another interpretation could be that these two eastern populations were formed by a large and diverse set of founders, and that this diversity has been simply maintained since founding. Without historical samples it may be difficult to differentiate between these two hypotheses, though future studies could sample recently colonized populations in the eastern US to estimate typical founding population sizes.

**Figure 6:**
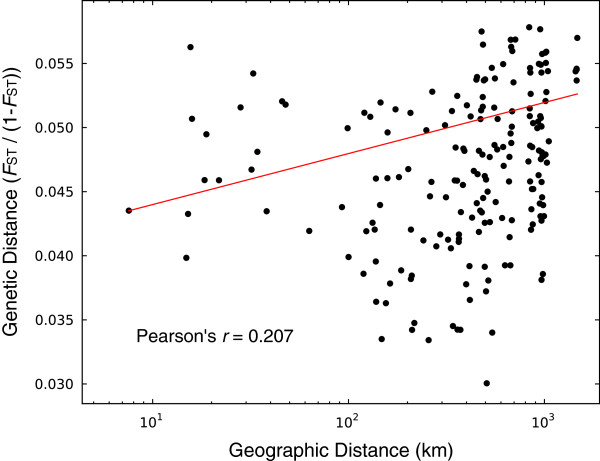
**Genetic isolation among WCR populations as a function of geographic distance.** Following Rousset [58], pairwise estimates of *F*_ST_ were linearized (*F*_ST_ /(1- *F*_ST_); *y*-axis) and regressed onto the natural log of the physical distance between populations (*x*-axis). The linear regression line is shown in red along with the Pearson’s correlation coefficient.

We do observe a modest – albeit significant – trend of increasing genetic isolation as a function of geographic distance among field collected populations (Figure 6; Pearson’s *r* = 0.207; Mantel test *p*-value = 0.002). Thus, there may be a subtle signature of the range expansion in the form of genetic isolation by distance. Moreover, the significance of the isolation by distance is strongly influenced by the inclusion of the most easterly population (Adams, IN). If we remove this population, the correlation between genetic and physical distance falls to 0.106 and is no longer significant (Mantel test *p*-value = 0.101). In contrast, if we remove all 5 populations from eastern Colorado and western Kansas (the most westerly populations) the correlation between genetic and physical distance is still significant (Pearson’s *r* = 0.256, Mantel test *p-*value = 0.003). The importance of this eastern population in detecting isolation by distance is consistent with incomplete homogenization following range expansion, however, because this result is driven by a single population we cannot exclude the hypothesis this population is in some way anomalous. Moreover, because recently formed Corn Belt WCR populations may not be at migration-drift equilibrium we cannot determine if the weak pattern of genetic isolation by distance is a product of genetic drift overcoming gene flow or if it is a hold-over from population founder effects during the species eastward invasion. Based on theory and these preliminary results we predict that sampling populations further east will tend to increase the extent of detectable of isolation by distance in WCR, and may reveal important estimates of genetic diversity in recently colonized areas.

In a review article, McDonald and Linde [59] posit that knowing information about the population genetics of a pest organism can lead to a better understanding of its evolutionary potential to evade control strategies. They proposed that species with high levels of genetic diversity and high gene flow between populations have the greatest evolutionary potential to overcome control options through Darwinian evolution. In this study, and in earlier studies [18, 21–25, 55], we find evidence to support the hypothesis that WCR has both high levels of genetic diversity and high gene flow between populations. For example, assuming WCR has a mutation rate (*μ*) between 10^-8^ or 10^-9^ bp^-1^ generation^-1^ (a range that encompasses estimates from *D. melanogaster* and *Caenorhabditis elegans*[60, 61]), the observed values of *π* imply that WCR field populations maintains approximate effective populations sizes (*N*_*e*_) on the order of 0.2 to 2 million individuals (i.e. *N*_*e*_ ≈ *π*/4 *μ*). This level of *N*_*e*_ gives little indication of recurrent population bottlenecks in WCR, which gives WCR populations the ability to generate and maintain many individuals with novel mutations. This creates a large pool of standing genetic variation that may in turn increase the probability of evolving resistance to control strategies. Moreover, we show very little genetic differentiation between field populations, and that genetic variation has spread quickly eastward following range expansion, both of which are consistent with high rates of gene flow between populations. Thus, if resistance does evolve, there are few barriers preventing it from spreading between populations. Together these two features of WCR population genetics are hypothesized to lead to a high evolutionary potential to evade controls [59], and may help to explain why WCR has a history of evolving resistance to insecticides and agricultural practices [3, 10].

## Conclusions

WCR is one of the most significant agricultural pests in North American, yet at the time of publication WCR has a modest number of nucleotide sequences are available in GenBank. Improved genetic and genomic resources for WCR are crucial tools in the ongoing effort to control this pest. Here we produce a large transcriptome assembly for WCR and complement these unigenes with diversity data obtained by whole genome population resequencing. Our WCR transcriptome assembly contains approximately 16,000 unigenes, including several gene families that have been implicated in insecticide resistance in other species. We identify a large pool of SNPs among 26 WCR populations, and show that these SNPs can be readily adapted for use as genotyping markers. We also use these SNPs to scan for outliers among candidate Bt resistance genes and to understand how population processes have shaped genetic variation in this species. We find two potential candidate Bt resistance genes that show strong patterns of molecular differentiation between high and low response to Cry3Bb1 populations. We also expand on past population genetic studies in WCR and show that this species has significant genetic diversity, little population structure, and a high evolutionary potential to resist control strategies. This study will provide a solid foundation for future research on the molecular genetics of WCR.

## Availability of supporting data

*454* ESTs and Illumina pooled population sequences used in this study can be found on the NCBI Sequence Read Archive under the accessions SRS528965 and SRP035322, respectively.

## Electronic supplementary material

Additional file 1: **GenBank IDs for public WCR EST sequences.** This spreadsheet contains the GenBank IDs for the public WCR sequences incorporated our transcriptome assembly. (XLSX 188 KB)

Additional file 2: **Population level WCR SNP genotypes.** A comma separated values file (csv) containing the genotypic status of all 26 populations sampled at 532,396 SNP sites. Missing genotypes are denoted by a dash. The sequence of each unigenes is listed on first usage. This file has been compressed with the bzip2 compression software. (ZIP 9 MB)

Additional file 3: **SNP genotyping validation study.** This spreadsheet contains the CAPs SNP validation information, including primer sequences, and observed restriction patterns and inferred genotypes. (XLSX 96 KB)

Additional file 4: **Power analysis of population genetic sampling procedure.** (DOCX 21 KB)

Additional file 5: **Blast2GO annotations.** This spreadsheet contains annotations for 10,359 WCR unigenes as identified by Blast2GO. (XLSX 1 MB)

Additional file 6: **Comparative genomics of WCR.** A Venn diagram showing the number of WCR contigs with significant matches (unique and common) to genomes of *T. casteneum*, *D. melanogaster*, *N. vitripennis*, and *B. mori*. The significant matches (*e* value < 10^-3^) were calculated after pairwise comparisons (BLASTx) to each individual genome. (PNG 77 KB)

Additional file 7: **Distribution and comparison of GO categories.** Vertical bars indicate the distribution of WCR (*Diabrotica*) and red flower beetle (*Tribolium*) GO term mappings that belong to each of the three top-level GO categories (i.e. biological process, molecular function, and cellular component). (PDF 47 KB)

Additional file 8: **KEGG pathway annotations.** This file contains two spreadsheets. The “Detailed” spreadsheet contains putative KEGG pathways assignments to unigenes in the WCR transcriptome, while the “Summary” spreadsheet lists a summary of the counts of all pathways identified. (XLSX 53 KB)

Additional file 9: **Identification of putative candidate genes for Cry3Bb1 resistance.** This file contains a spreadsheet for each of several putative gene families that may be candidates for resistance to Bt toxins, including the unigenes name and descriptions, and results from *π*_*xy*_and *F*_*ST*_ outlier scans between Cry3Bb1 resistant and susceptible populations. The last two spreadsheets list the results of *π*_*xy*_and *F*_*ST*_ outlier scans between populations with high or low response to Cry3Bb1 for all available unigenes. (XLSX 4 MB)

Additional file 10: **Characterization of WCR-CYP genes.** BLASTp result comparing WCR-CYP unigenes to a *Tribolium* CYP data set [18]. (XLSX 17 KB)

Additional file 11: **Identification of candidate genes for non-diapause trait.** This spreadsheet contains the results of *π*_*xy*_outlier scans between diapause and non-diapause populations for all available unigenes. Along with the scan results, unigenes name and descriptions are also given. (XLSX 2 MB)

Additional file 12: **Plot of calculated** ***π***_***xy***_ **values between diapause and non-diapause populations and permuted 99** ^**th**^ **percentile values.** Each point represents a unigenes calculated *π*_*xy*_ value and the 99^th^ percentile value from 1,000 permutations of the diapause and non-diapause population labels. Red dots indicate calculated *π*_*xy*_ > 99^th^ percentile permutation value (outliers), while black dots represent calculated *π*_*xy*_ < 99^th^ percentile permutation value. The blue line represents the boundary between outliers and non-outliers. (PDF 1 MB)

Additional file 13: **A histogram of pairwise** ***F***_**ST**_ **between WCR populations.** This histogram represents *F*_ST_ estimates for all genes among all pairwise comparisons of the 26 WCR populations. (PDF 76 KB)
