# Supplementary material for: Western corn rootworm (Diabrotica virgifera virgifera) transcriptome assembly and genomic analysis of population structure
Source: BMC Genomics. 2014 Mar 14;15(1):195. doi: 10.1186/1471-2164-15-195 (PMC4004143; doi:10.1186/1471-2164-15-195)
Supplement: Supplementary file 4 — Additional file 4: Power analysis of population genetic sampling procedure. (DOCX 21 KB) [file 12864_2013_7033_MOESM4_ESM.docx]

POPULATION STUDY POWER ANALYSIS

In this study the approach we employed was to first sample 5 randomly collected diploid individuals from the population, and then using Illumina sequencing, sample their 10 alleles on average 20-25 times. Below we outline several analyses that help characterize the statistical power of our approach for estimating population parameters.  Specifically, because all population genetic estimators used in this study (π, FST, and $\pi_{xy}$) operate on allele frequencies, we describe the expected error in allele frequency estimates. We have broken the problem into 3 pieces.

First, we can describe the error we should expect when sampling 10 alleles to estimate the allele frequency for a population.  For segregating sites among 10 alleles there are 9 possible allelic states.  **Table S1** list each below in terms of arbitrary *A* and *a* alleles:

**Table S1**

| *A* | *a* |
| --- | --- |
| 1 | 9 |
| 2 | 8 |
| 3 | 7 |
| 4 | 6 |
| 5 | 5 |
| 6 | 4 |
| 7 | 3 |
| 8 | 2 |
| 9 | 1 |

Given these states, a natural way to model the expected allele frequency error is using the beta distribution. **Table S2** below uses the beta distribution to describe various aspects of the expected error of our estimate with respect to the unknown true allele frequency in the population. For each of the 9 allelic states **Table S2** gives the observed allele frequency of *A*, the upper and lower 95% confidence intervals of this estimate with respect to the true population allele frequency, and the root-mean-square error (RMSE), which is the expected mean standard deviation between the observed value and the real population value.

**Table S2**

| *A* | *a* | obs. *A* freq. | *A* freq. lower 95% bound | *A* freq. upper 95% bound | *A* freq. RMSE |
| --- | --- | --- | --- | --- | --- |
| 1 | 9 | 0.1 | 0.00 | 0.34 | 0.09 |
| 2 | 8 | 0.2 | 0.03 | 0.48 | 0.12 |
| 3 | 7 | 0.3 | 0.07 | 0.60 | 0.14 |
| 4 | 6 | 0.4 | 0.14 | 0.70 | 0.15 |
| 5 | 5 | 0.5 | 0.21 | 0.79 | 0.15 |
| 6 | 4 | 0.6 | 0.30 | 0.86 | 0.15 |
| 7 | 3 | 0.7 | 0.40 | 0.93 | 0.14 |
| 8 | 2 | 0.8 | 0.52 | 0.97 | 0.12 |
| 9 | 1 | 0.9 | 0.66 | 1.00 | 0.09 |

From **Table S2** we can conclude that a sample size of 10 alleles is adequate for genetic diversity and differentiation calculations.  For example, if you were to genotype 5 diploid individuals at a site and find two C nucleotides and eight Ts, 95% of the time you’d expect the true population frequency of C to be between 0.03 and 0.48, and half the time it would be between 0.11 and 0.27 (not shown).  That is, the estimate of 0.2 would be within +/- 0.09 half the time. The RMSE offers another way to think of this in terms of standard deviates. The observed value +/- one standard deviate would be 0.2 +/- 0.12.

Moreover, the RMSE for alleles among 20 diploid individuals found at C=8 and T=32 is 0.062 (data not shown) and the 95% confidence interval ranges from 0.09 to 0.34.  This represents a four-fold sampling increase to 20 diploid individuals (a value often found in population studies), and only roughly halves the RMSE and similarly marks only a modest reduction in the 95% confidence interval.

Next we need to consider the impact of sampling the 9 possible allelic states using sequencing.  To do this we have used the observed data from 10,359 unigenes, which encompasses over 300,000 segregating sites across all 26 populations.  Following sequencing, each segregating site within a population has an observed state, say for example 4 Cs and 6 Ts.  The estimated population allele frequency for C at this site is 0.4.  This is the same estimate as a different site which has 8 Cs and 12 Ts, despite this latter site having twice the sequence coverage.  For simplicity we binned all segregating sites into 5 classes on the basis of their estimated minor allele frequency (MAF) (i.e MAF bins of (0-0.1], (0.1-0.2], (0.2-0.3], (0.3-0.4], and (0.4-0.5]).  Then for each bin we again estimated the RMSE using a beta distribution, which in each case takes into account the empirical distribution of sequence coverage.  **Table S3** lists the result for the Hopkinton population, which we will use as an exemplar.  Due to comparable relative diversity and sequencing effort for each population, results from all populations look similar.  Also, note that we’ve eliminated the major allele from **Table S3**.  Just as in the table above the estimates are symmetrical around 0.5, so it is unnecessary to consider both the major and minor allele.

**Table S3**

| bin | lower freq. bound | upper freq. bound | sites | RMSE |
| --- | --- | --- | --- | --- |
| 1 | 0 | 0.1 | 53,009 | 0.05 |
| 2 | 0.1 | 0.2 | 20,586 | 0.10 |
| 3 | 0.2 | 0.3 | 10,836 | 0.13 |
| 4 | 0.3 | 0.4 | 8,065 | 0.13 |
| 5 | 0.4 | 0.5 | 6,604 | 0.14 |

As before, the RMSE is fairly low.  Also, as before, the RMSE is lowest when the minor allele is rare, and highest when the minor and major alleles are both near 0.5.  Note that most of the sites have one rare and one common allele, the condition under which the RMSE is lowest.  This means that most sites get estimates with the lowest possible variance given the experimental design.  And similar to before, quadrupling sequence effort (i.e. 80-100X coverage) would only reduce RMSE by about a half (not shown).

We can pull both sampling processes together in an effort to characterize the expected error between a true population parameter and an estimate of this parameter made by randomly sampling 5 individuals and sequencing them to 25X coverage.  In this case the parameter we will focus on is the expected heterozygosity (*He*), since this forms the basis of estimating π, FST, and $\pi_{xy}$.  Here for simplicity we have abandoned the analytical approach used above and have switched to a Monte Carlo simulation.  Briefly, we choose a “true” MAF, then we make 100,000 random samples of size 10 (the number of haploid genomes used in our study) from a binomial distribution with probability (p) equal to the true MAF.  Next we resample each of the 100,000 random draws 25 times (approx. the expected mean sequence coverage), again using a binomial distribution, which in each case in conditioned with the probability (p) equal to the realized MAF from the earlier random sample of size 10.  Finally we calculate *He* for this random selection and compare this result to the true *He*.  This approach does a good job of characterizing the error from end-to-end for our experimental design.  **Table S4** outlines the results of the Monte Carlo simulation.

**Table S4**

| true MAF freq. | true *He* | *He* RMSE (5 indv.; 25X seq. cov.) | *He* RMSE (20 indv.; 100X seq. cov.) |
| --- | --- | --- | --- |
| 0.05 | 0.095 | 0.13 | 0.08 |
| 0.1 | 0.18 | 0.16 | 0.11 |
| 0.15 | 0.255 | 0.18 | 0.13 |
| 0.2 | 0.32 | 0.18 | 0.14 |
| 0.25 | 0.375 | 0.17 | 0.14 |
| 0.3 | 0.42 | 0.15 | 0.13 |
| 0.35 | 0.455 | 0.13 | 0.11 |
| 0.4 | 0.48 | 0.10 | 0.08 |
| 0.45 | 0.495 | 0.09 | 0.04 |
| 0.5 | 0.5 | 0.11 | 0.03 |

In **Table S4** we supplied 10 true MAFs and calculated the expected RMSE for *He* each under the sampling regime we used (5 indv.; 25X seq. cov.), and for another regime which is four times larger than ours (20 indv.; 100X seq. cov.).  As before quadrupling our sampling only makes a fractional reduction to the RMSE. In fact, the RMSE is more influenced by the allele frequency (a parameter that cannot be controlled) than it is by choice of sampling regime.

Finally, all the results above apply to a single SNP site.  We pool across SNPs to get an estimate for each unigene and then use all available unigenes to get the grand mean values of π, FST, and $\pi_{xy}$.  These grand mean estimates involve ~10,000 unigenes and ~100,000 SNP sites for each population or population contrast.  Given these large sample sizes, the law of large numbers strongly suggest that our grand means are accurate estimators of the true underlying parameters regardless of the variance of each individual SNP.
