# Supplementary figures and images for: Western corn rootworm (Diabrotica virgifera virgifera) transcriptome assembly and genomic analysis of population structure
Source: BMC Genomics. 2014 Mar 14;15(1):195. doi: 10.1186/1471-2164-15-195 (PMC4004143; doi:10.1186/1471-2164-15-195)

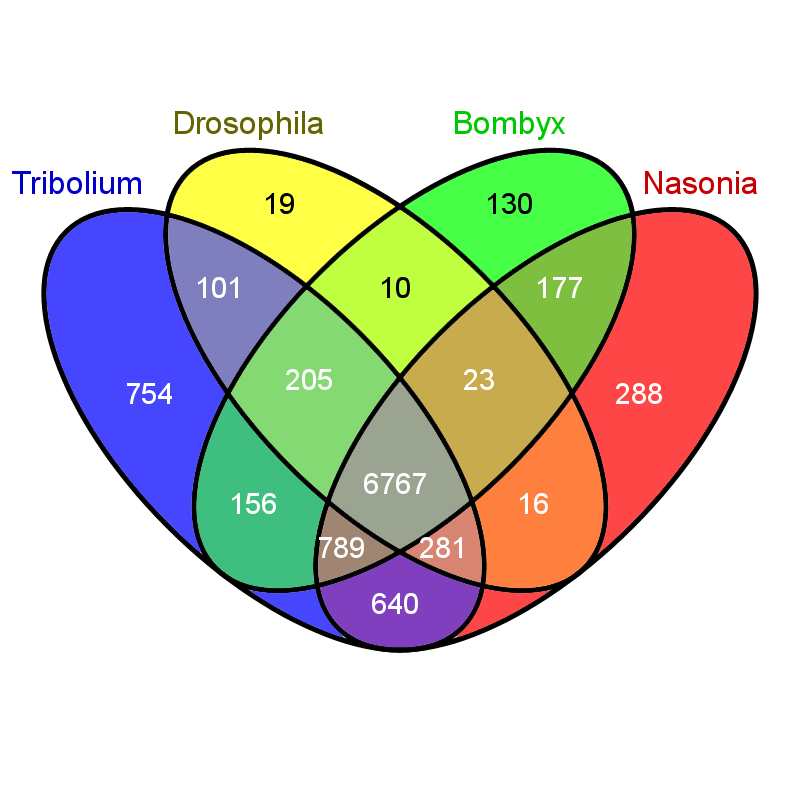

Supplement: Supplementary file 6 — Additional file 6: Comparative genomics of WCR. A Venn diagram showing the number of WCR contigs with significant matches (unique and common) to genomes of T. casteneum, D. melanogaster, N. vitripennis, and B. mori. The significant matches (e value < 10-3) were calculated after pairwise comparisons (BLASTx) to each individual genome. (PNG 77 KB) [file 12864_2013_7033_MOESM6_ESM.png]

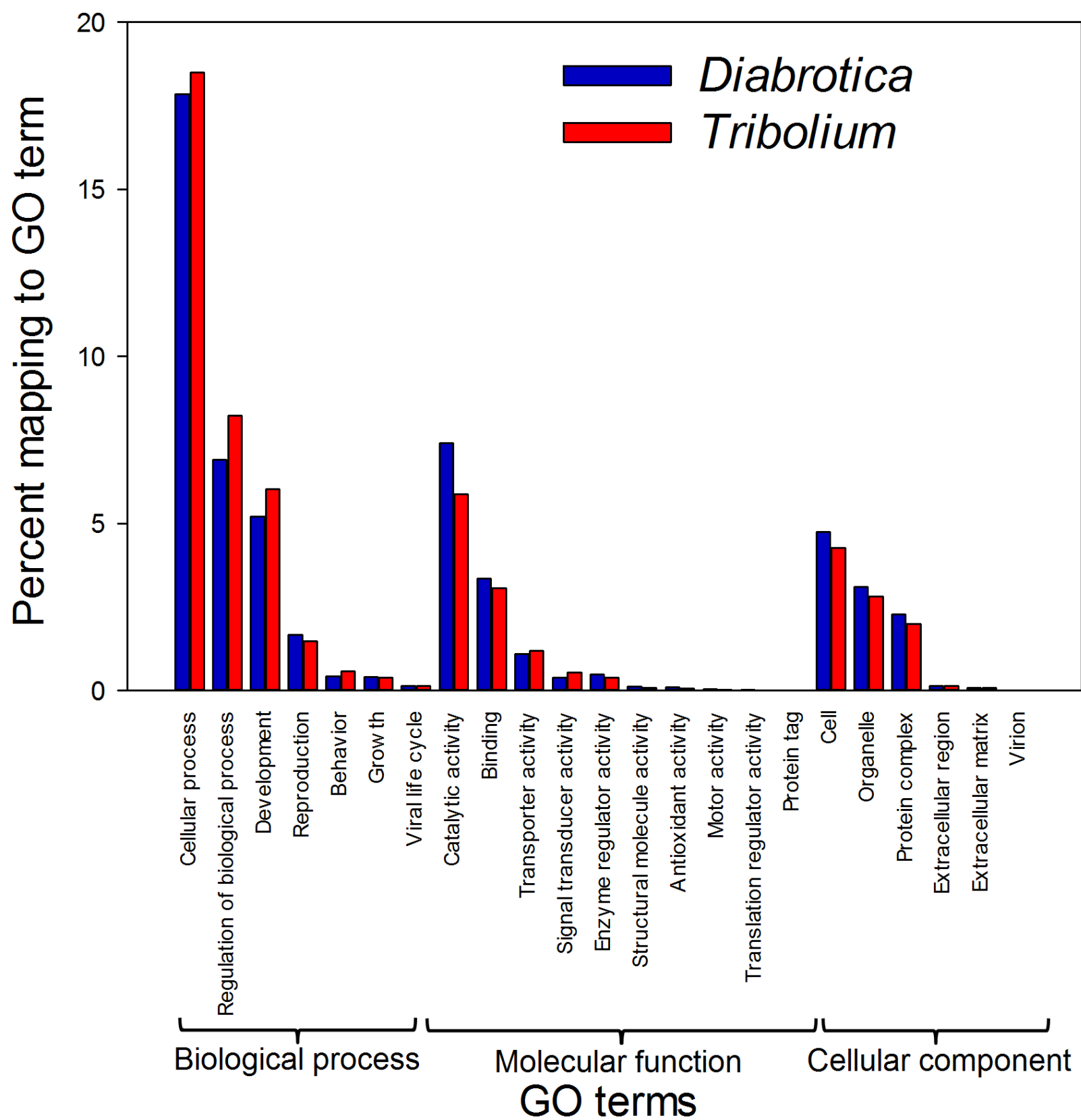

Supplement: Supplementary file 7 — Additional file 7: Distribution and comparison of GO categories. Vertical bars indicate the distribution of WCR (Diabrotica) and red flower beetle (Tribolium) GO term mappings that belong to each of the three top-level GO categories (i.e. biological process, molecular function, and cellular component). (PDF 47 KB) [file 12864_2013_7033_MOESM7_ESM.pdf]

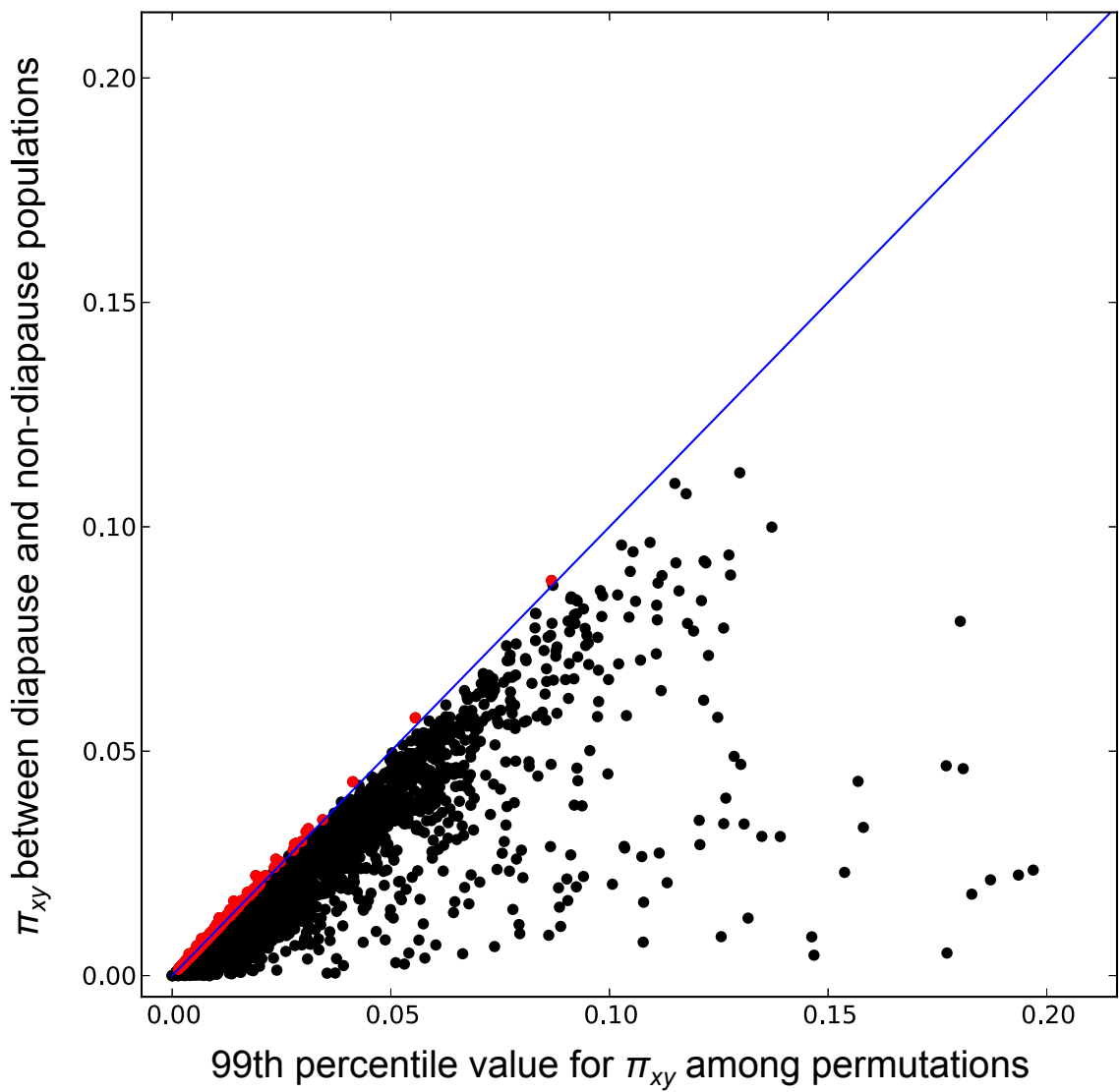

Supplement: Supplementary file 12 — Additional file 12: Plot of calculated π xy values between diapause and non-diapause populations and permuted 99 th percentile values. Each point represents a unigenes calculated π xy value and the 99th percentile value from 1,000 permutations of the diapause and non-diapause population labels. Red dots indicate calculated π xy > 99th percentile permutation value (outliers), while black dots represent calculated π xy < 99th percentile permutation value. The blue line represents the boundary between outliers and non-outliers. (PDF 1 MB) [file 12864_2013_7033_MOESM12_ESM.pdf]

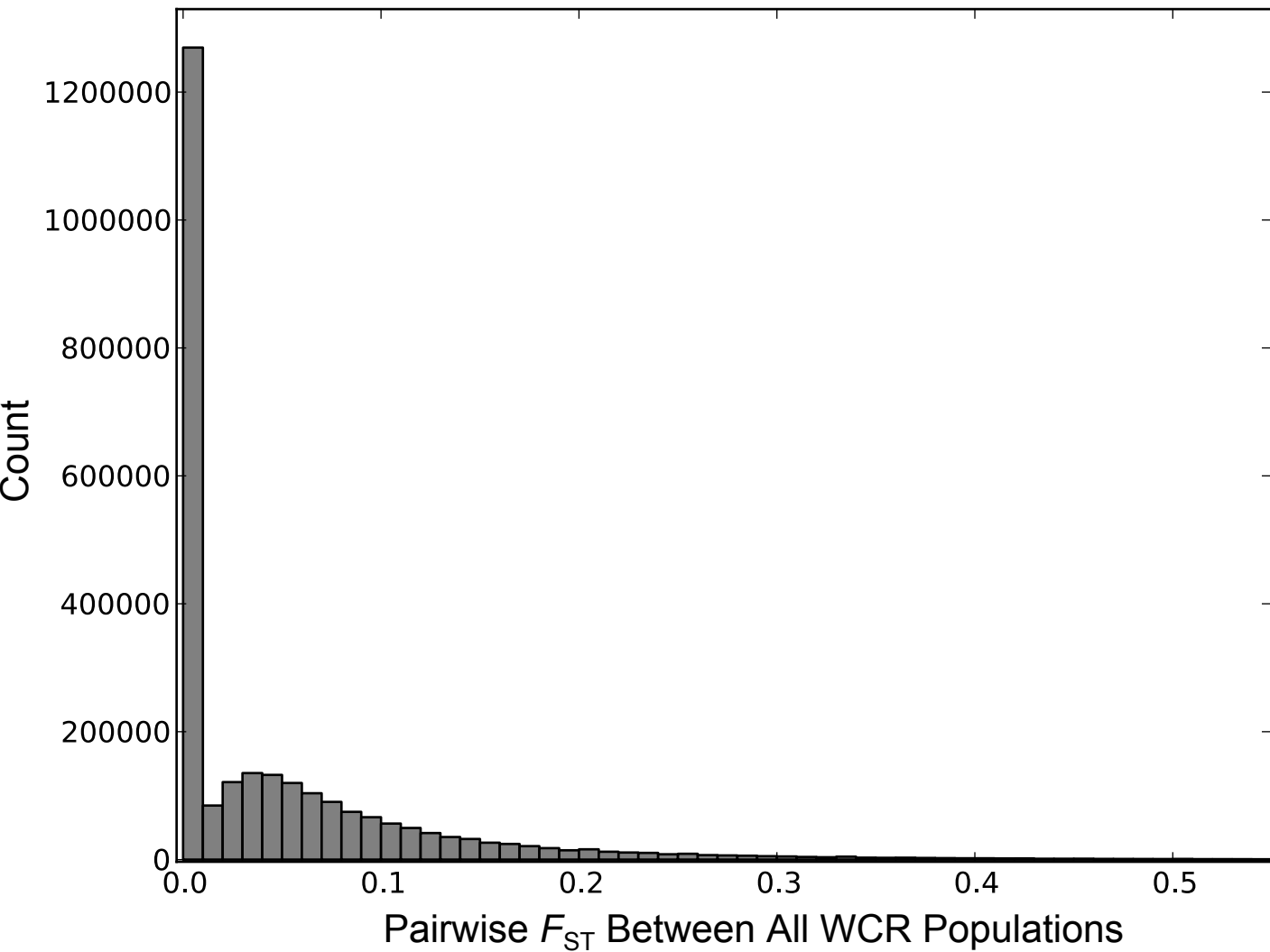

Supplement: Supplementary file 13 — Additional file 13: A histogram of pairwise F ST between WCR populations. This histogram represents F ST estimates for all genes among all pairwise comparisons of the 26 WCR populations. (PDF 76 KB) [file 12864_2013_7033_MOESM13_ESM.pdf]
